# Hereditary hemorrhagic telangiectasia in a sudanese patient: A case report

**DOI:** 10.1002/ccr3.5585

**Published:** 2022-03-13

**Authors:** Fadi M. Toum Ahmed, Moh. Mah. Fadelallah Eljack, Mohamed Abdulkarim, Hiba Faroug Alamin Mohammed

**Affiliations:** ^1^ Internal Medicine Specialist Sinnar University Sennar Sudan; ^2^ Faculty of Medicine and Health Sciences University of Bakht Alruda Aldueim Sudan; ^3^ Alzaiem Alazhari University Khartoum Sudan; ^4^ Registrar of internal medicine Sennar Sudan

**Keywords:** bleeding, colitis, epistaxis, hereditary, sudanese, telangiectasia

## Abstract

(HHT) is a rare disorder affecting the skin and body's internal organs with a tendency for bleeding. We report a case of Sudanese 42‐year‐old with family history of HHT presented with recurrent epistaxis and telangiectasias.

## Graphical abstract

Any patient presents frequently with abnormal bleeding should be evaluated properly from digging deeply in their own and their family history, detailed examination, and investigations starting with routine up to invasive one.

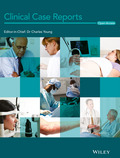

## 1 INTRODUCTION

Hereditary hemorrhagic telangiectasia (HHT) (also known as Osler‐Weber‐Rendu syndrome (OWRS)) is a rare dominant autosomal disorder with a frequency of 6.1–12.1 per 100,000.^1^ There are no differences between genders. It is clinically characterized by telangiectasia, recurrent epistaxis, and visceral vascular lesions (arteriovenous malformations—AVMs). Usually, a person with HHT has a family history of the disorder. This article reports a case that is clinically compatible with this rare entity.

## 2 PATIENT INFORMATION

Our patient is a 42‐year‐old man who presented with a lifelong bleeding problem. On admission, he was concerned about an extensive bleeding through his nose (right nostril). Since he was 11‐year‐old, he presented with frequent attack of epistaxis. When his age was 27 years, he presented with epistaxis of large amount, investigations showed a very low platelets (15*10)3, MCV = 83 f.l, Bone Marrow aspirate showed cellular reactive suggests that thrombocytopenia mostly due to peripheral removal. He received 6 units of blood. A diagnosis of idiopathic thrombocytopenic purpura was made for which he put on corticosteroids and osteoprotection, with no improvement of his condition. He also takes proton pump inhibitors and tranexamic acid to help reduce the bleeding. In 2017, when his age was 37, he presented with massive upper and lower GI bleeding for which he underwent endoscopy that showed gastric and duodenal ulcers and colonoscopy showed hyperemic mucosa, sever inflammation, and colonic polyps; a diagnosis of ulcerative colitis was made and he putted on mesalazine and steroids for three months and also without significant improvement.

He has two family members with the same bleeding problem including a brother and a sister. They were already diagnosed with HHT and passed away due to refractory HF, DCM, AF, and massive blood loss. He is not known to have any allergies and has never received any drug that might interfere with blood homeostasis.

## 3 EXAMINATION

**FIGURE 1:**
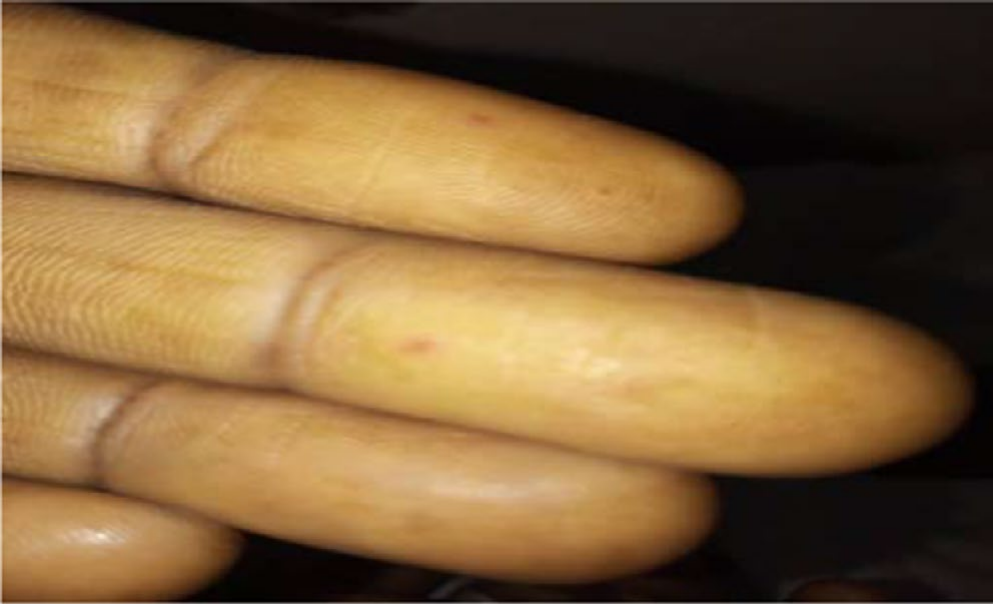
Fingertip telangiectasias

**FIGURE 2:**
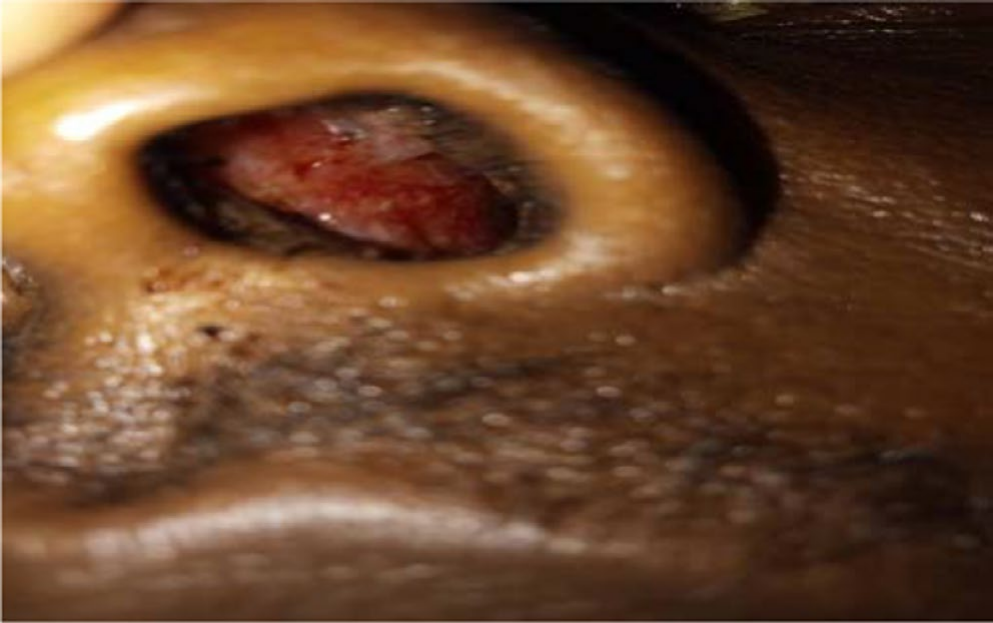
Mucosal hyperemia and telangiectasia

**FIGURE 3:**
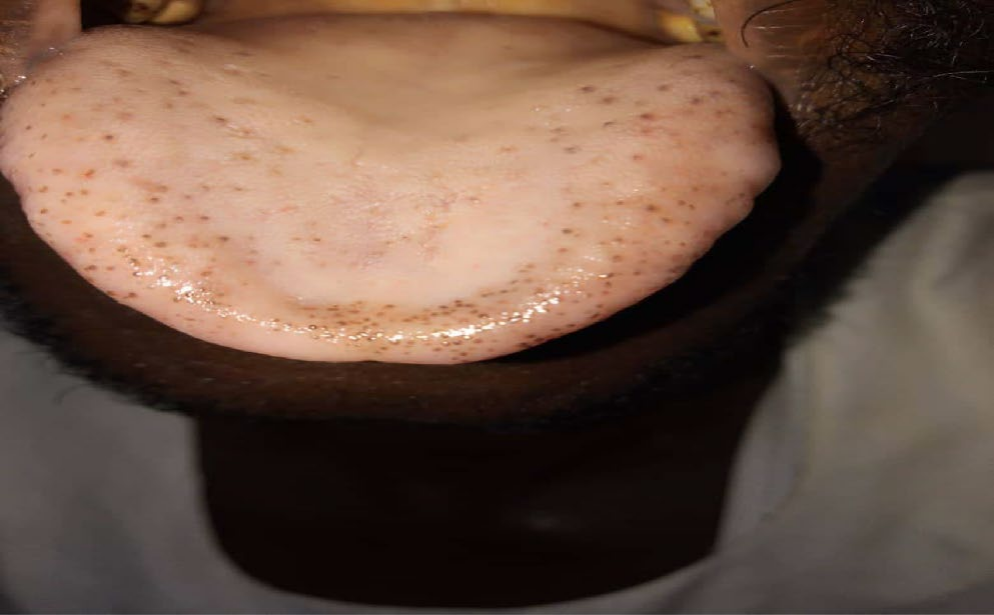
Tongue telangiectasia

Examination revealed a fully conscious patient (GCS = 15), pulse was 80 beat per minute, and blood pressure was 100/65 mmhg. Upon examining his skin and mucus membrane multiple purpuric, punctuate, tiny macules in palms and fingertips of both hands were noted (Figure 1). These macules also appeared on the oral and nasal mucosae and on the tongue (Figures 2 and 3). His cardiac examinations revealed normal first and second heart sounds with pansystolic functional murmur. When examining his abdomen, his liver and spleen were palpable (10 and 6 cm below the costal margin, respectively), with normal other measures.

## 4 INVESTIGATIONS

Blood tests showed hemoglobin = 8 g/dl, mean corpuscular volume = 86.6fl, platelets = (396* 10)3 normal WBCS, and retics count = 6.2, ESR = 20. Renal function test + electrolytes, liver function test were all normal. Unfortunately, we found him to be HCV positive, and we owe that to the frequent blood transfusions which he received.

Other iron parameter tests were not available in the city and the patient refuse to be referred.

The abdominal ultrasound confirm hepatosplenomegaly (with no focal lesions), echocardiography was normal.

Based on the presence of epistaxis, telangiectasias and a first‐degree relative with HHT confirmed using the Curacoa criteria.^2^

## 5 TREATMENT AND FOLLOW‐UP

With regard to the treatment this patient received, it was mainly supportive in nature since no cure has been found till now. The patient was treated through nasal packing for his epistaxis along with tranexamic acid. While we were not able to do contrast‐enhanced pulmonary CT scan looking for pulmonary AVMs, it is usually managed with embolization. The patient was followed up in the referred clinic for a period of 3 months with no significant complications apart from his epistaxis.

## 6 DISCUSSION

Hereditary hemorrhagic telangiectasia (HHT), also known as Osler‐Weber‐Rendu disease is a rare autosomal dominant (AD) vascular disorder affecting the skin and body's internal organs in which there is an increased tendency for bleeding.

A consensus upon diagnostic testing is applied as Curacoa criteria which include 2 criteria out of 4 for possible diagnosis and 3 out of 4 criteria for confirming the diagnosis. These criteria include recurrent episodes of epistaxis, telangiectasias in the skin and mucosal surfaces, arteriovenous malformations (AVMs) in visceral organs (lung, liver, brain, and spine) and having a first‐degree relative with confirmed disease using the same criteria.^3^

There are not many cases reported from the African continent with a case of a 60‐year‐old African woman from Nairobi, Kenya, has been described.^4^ We think that the reason might be that diagnosing such a condition can be challenging in low‐resource countries such as Sudan in this case. Availability of complex investigation modalities away from big cities can be a big hindrance in terms of diagnostic approach to patients with suspected HHT.

In this article, we report a case of a 42‐year‐old Sudanese man who presented with a lifelong bleeding problem with recurrent epistaxis and known first‐degree relative (a brother) with HHT diagnosed using the same Curacoa criteria who has died. He has multiple telangiectasias in his palm skin. His colonoscopy revealed no telangiectasia, though. Due to financial difficulties and availability investigations modalities, the patient was not able to do vascular imaging studies to look for AVMs in lungs, liver, or brain. He was found to be positive for hepatitis C virus, which we suggest that it could be related to recurrent transfusions he received due to his anemia from chronic blood loss. While hepatomegaly has been reported in the case of the Kenyan woman, this patient has enlargement of not only his liver but also his spleen (10 and 6 cm below the costal margin, respectively), along with visible purpuric rash. In this article, we aimed to expand the literature about this disease in Africa.

Recurrent epistaxis is the single most common manifestation of HHT that is affecting up to nearly 90% of patients.^5^ It can be massive and progress to an emergency affecting the hemodynamic stability of patients. It is usually managed and controlled by nasal packing and tranexamic acid. Recurrent bleeding results in anemia that can be managed using blood transfusion and iron replacement therapies. While this patient has the predominant mucocutaneous manifestations, visceral organs involvement can lead to some serious complications including hypoxemia from secondary shunting of the blood due to pulmonary AVMs and possible paradoxical pulmonary embolism for the mechanism which was reported in the literature.^5^ Strokes, both ischemic and hemorrhagic have also been reported.

## 7 CONCLUSION

Although of its rare incidences; hereditary hemorrhagic telangiectasia should be kept in mind for patients present with spontaneous recurrent epistaxis especially when the patient has a positive family history, a simple approach is to search for telangiectasia within skin and mucous membranes. However, this faced by scare of resources to search for internal sites involvement.

When diagnosis is made hemoglobin should be followed closely to prevent fatal complications of anemia.

## CONFLICTS OF INTEREST

The authors report no conflict of interest.

## AUTHOR CONTRIBUTIONS

All authors participated in planning the study. FMT and MME collected data, did investigations and examination, and wrote the first draft. M.A and H.F.A supervised the process of the study and revised the draft. All authors participated significantly in writing the draft.

## ETHICAL APPROVAL

Ethics approval was obtained from Ethical committee at University of Sinnar and informed consent was taken from the patient for purposes of publications.

## CONSENT

Written informed consent was obtained from the patient for publication of this case report and accompanying images.

## Data Availability

The data that support the findings of this study are available with corresponding author upon reasonable request.
